# Editorial: Advancements in chemical biology

**DOI:** 10.3389/fchem.2026.2014579

**Published:** 2026-10-13

**Authors:** John D. Wade

**Affiliations:** 1 Florey Institute of Neuroscience and Mental Health, University of Melbourne, Parkville, VIC, Australia; 2 School of Chemistry, University of Melbourne, Parkville, VIC, Australia

**Keywords:** antioxidant, bioconjugates, chemical biology, deSUMOylation, gold(I)-NHC complexes, *in situ* activated warheads, LC-MS, lipidated peptides

Chemical biology has matured into a highly interdisciplinary field that seeks not only to understand biological systems at the molecular level, but also to manipulate them with increasing precision. By integrating advances in chemical synthesis, recombinant technologies, biomolecular engineering, structural biology, proteomics, and analytical sciences, the discipline provides powerful tools for exploring complex biological processes and translating fundamental discoveries into practical applications.

In this Research Topic, seven contributions illustrate the breadth and impact of contemporary chemical biology. Collectively, they demonstrate how molecular insight can be converted into new technologies, mechanistic understanding, and therapeutic opportunities spanning neuroscience, natural products, medicinal chemistry, peptide science, antimicrobial research, and biomaterials.

Xu et al. combined molecular modelling, peptide design, and animal studies to investigate the regulation of neuronal nitric oxide synthase (nNOS). By integrating computational and experimental approaches, they identified K739 as a key regulatory site exhibiting preferential SENP1-mediated deSUMOylation and showed that engineered CaM-binding domain peptides can influence neuroprotective outcomes following stroke. This work exemplifies the ability of chemical biology to connect molecular mechanisms with physiological function and therapeutic potential.

Kerroum et al. explored the medicinal plant Rheum officinale through detailed phytochemical and biological analyses. Their findings identified quinic acid as a major constituent and revealed significant antioxidant, antimicrobial, and enzyme-inhibitory activities, highlighting the continuing importance of natural products as a source of biologically active molecules and potential therapeutic leads.

Giorgi et al. reported a series of gold(I)-NHC complexes designed for anticancer applications. Structural modifications, particularly thiosugar conjugation, were shown to improve cellular uptake, potency, and selectivity in ovarian cancer models. The study illustrates how molecular design, bioconjugation, and biological evaluation can be integrated to advance targeted cancer therapeutics.

In a complementary review, Jain and Iwaoka examined recent progress in covalent inhibitors that target cysteine and selenocysteine residues. Their article highlights both established and emerging warhead chemistries and discusses the opportunities and challenges associated with achieving selectivity, reducing toxicity, and improving *in vivo* stability. These developments underscore the growing importance of covalent strategies in precision medicine.

Taking a different perspective, Roque-Borda et al. examined peptide lipidation and its influence on biological activity. They argue that improvements in potency frequently arise from altered membrane interactions, distribution, and residence time rather than enhanced intrinsic target engagement. Their analysis provides an important framework for the mechanistic interpretation and rational design of lipidated peptides.

Two contributions focus on antimicrobial resistance, an area in which chemical biology continues to make important advances. Baravkar et al. reviewed antimicrobial depsipeptides and cyclodepsipeptides, demonstrating how subtle structural features govern interactions with bacterial membranes and biosynthetic pathways. These macrocyclic peptides remain valuable molecular probes and promising scaffolds for antibiotic development.

The final contribution, by Wilde et al., addresses antimicrobial materials inspired by natural antimicrobial peptides. By examining the interactions of polymer-based materials with bacterial membranes, the authors provide mechanistic insight that supports the rational development of next-generation antimicrobial surfaces and coatings for healthcare and community settings.

Taken together, the articles collected in this Research Topic underscore the breadth and vitality of modern chemical biology. Spanning neurobiology, natural products, medicinal chemistry, peptide science, antimicrobial research, and biomaterials, they demonstrate how molecular-level investigation continues to generate fundamental biological insight while inspiring new therapeutic and technological opportunities. It is anticipated that this Research Topic will stimulate further advances across the many disciplines that contribute to, and benefit from, contemporary chemical biology.

